# Advances in Regenerative Medicine and Tissue Engineering: Innovation and Transformation of Medicine

**DOI:** 10.1155/2018/2495848

**Published:** 2018-07-30

**Authors:** Kevin Dzobo, Nicholas Ekow Thomford, Dimakatso Alice Senthebane, Hendrina Shipanga, Arielle Rowe, Collet Dandara, Michael Pillay, Keolebogile Shirley Caroline M. Motaung

**Affiliations:** ^1^Cape Town Component, International Centre for Genetic Engineering and Biotechnology (ICGEB) and UCT Medical Campus, Wernher and Beit Building (South), Anzio Road, Observatory 7925, Cape Town, South Africa; ^2^Division of Medical Biochemistry and Institute of Infectious Disease and Molecular Medicine, Department of Integrative Biomedical Sciences, Faculty of Health Sciences, University of Cape Town, Anzio Road, Observatory 7925, Cape Town, South Africa; ^3^Pharmacogenetics Research Group, Division of Human Genetics, Department of Pathology and Institute of Infectious Diseases and Molecular medicine, Faculty of Health Sciences, University of Cape Town, Observatory 7925, Cape Town, South Africa; ^4^Department of Biotechnology, Faculty of Applied and Computer Sciences, Vaal University of Technology, Vanderbijlpark 1900, South Africa; ^5^Department of Biomedical Sciences, Faculty of Science, Tshwane University of Technology, Pretoria 0001, South Africa

## Abstract

Humans and animals lose tissues and organs due to congenital defects, trauma, and diseases. The human body has a low regenerative potential as opposed to the urodele amphibians commonly referred to as salamanders. Globally, millions of people would benefit immensely if tissues and organs can be replaced on demand. Traditionally, transplantation of intact tissues and organs has been the bedrock to replace damaged and diseased parts of the body. The sole reliance on transplantation has created a waiting list of people requiring donated tissues and organs, and generally, supply cannot meet the demand. The total cost to society in terms of caring for patients with failing organs and debilitating diseases is enormous. Scientists and clinicians, motivated by the need to develop safe and reliable sources of tissues and organs, have been improving therapies and technologies that can regenerate tissues and in some cases create new tissues altogether. Tissue engineering and/or regenerative medicine are fields of life science employing both engineering and biological principles to create new tissues and organs and to promote the regeneration of damaged or diseased tissues and organs. Major advances and innovations are being made in the fields of tissue engineering and regenerative medicine and have a huge impact on three-dimensional bioprinting (3D bioprinting) of tissues and organs. 3D bioprinting holds great promise for artificial tissue and organ bioprinting, thereby revolutionizing the field of regenerative medicine. This review discusses how recent advances in the field of regenerative medicine and tissue engineering can improve 3D bioprinting and vice versa. Several challenges must be overcome in the application of 3D bioprinting before this disruptive technology is widely used to create organotypic constructs for regenerative medicine.

## 1. Introduction

Tissue and organ shortages have been identified as a major public health challenge with only a small percentage of deserving patients receiving transplantations [1, 2]. Most waiting lists for tissues and organs do not capture the magnitude of the crisis well as only those who are sick seek such assistance [3–8]. The terms regenerative medicine and tissue engineering are used with appreciable overlap by scientists and clinicians and in this review are used as synonyms. The promise of regenerative medicine is founded on the potential and ability to regenerate and replace damaged tissues and organs [9, 10]. Regenerative medicine has shown promising results for the regeneration and replacement of a variety of tissues and organs including skin, heart, kidney, and liver and the potential to even correct some congenital flaws [11–13]. The traditional reliance on donated tissues and organs for transplantations faces the problem of donor shortages and possible immunological rejection of the donated body parts [14, 15]. Some of the organ transplants performed in developing nations include cases of transplant tourism where foreigners, with enough money and influence, are given priority over the local populace [1, 16, 17]. Such practices have been condemned as it can result in the exploitation of defenseless people [1, 18, 19]. Despite differences in national economic powers and therefore differences in healthcare infrastructure, overcoming burdens such as the low supply of organs and the practical hurdles of collecting and storing them can help in increasing the number of people who can undergo organ transplantations [1, 20, 21]. Therefore, strategies and technologies that can increase the supply of tissues and organs for transplantation must be developed further. In most cases, tissues and organs are required immediately for transplantation as is the case when people are wounded in accidents, wars, and natural disasters [22, 23]. The shortage of tissues and organs not only hampers the treatment of patients but also hinders scientific research. The development of an endless supply of tissues and organs therefore represents the most challenging task of our generation. Many initiatives have been undertaken to increase organ donations and better usage of the donated organs [24–26]. One solution is the advent of laboratory-grown tissues, humanized animal organs, and bioartificial organs [27, 28]. Regenerative medicine may help in solving some of these challenges [29, 30].

For regenerative medicine strategies to be successful, the material used, mostly combinations of scaffolds, growth factors, and stem cells, must be able to replace the damaged tissue and be able to function as the original tissue or be able to stimulate regeneration of the original tissue [31, 32]. Cells used in regenerative medicine and tissue engineering can come from the same patient (autologous) or from another individual (allogeneic). In addition, xenogenic cells such as those from animals can also be adopted in regenerative medicine strategies. Cells that have been used so far include stem cells, fibroblasts, chondrocytes, and keratinocytes [33, 34]. Though allogeneic cells might illicit an immune reaction, this can be alleviated by prescribing immunosuppressants to patients. Depending on the age of the patient, some regenerative medicine strategies can utilize and accelerate the body's own natural healing process [35, 36]. These strategies are aimed at changing the tissue environment by the introduction of exogenous material and biological factors with the sole aim of accelerating and improving the body's healing process. Materials and biomimetics of the extracellular matrix have been in use for several years now and do more than just providing the physical structure [37–39]. Materials and biomimetics can stimulate regeneration on their own but can also be used to present biomolecules such as growth factors to promote the growth of cells [32, 34, 38–40]. Initially thought to be necessary for physical support for cells, the biomaterial or scaffold can now incorporate biological cues or signals to enhance or promote tissue regeneration and function [41–43]. Due to the different regeneration capacities of different tissues, some tissues may not require cells but just the biomaterial and biologics whilst other tissues have limited regeneration capacities and require the biomaterial, biomolecules, and cells for regeneration to occur. Tissues and organs with limited or no regeneration capacity at all include the cartilage and cornea whilst those with high regeneration capacities include the liver and the lungs [9, 44, 45].

Several 3D-bioprinted constructs and stem cell therapies have been approved by the Food and Drug Administration (FDA) and the European Medicines Agency (EMA) in the last 10 years [11, 12, 36, 46]. These therapies and products range from biologics and medical devices to biopharmaceuticals [36, 47, 48]. Biomolecules and growth factors can be tethered to the materials and can provide sustained stimuli to promote cellular differentiation and regeneration of damaged tissue. Growth factors that have been used this way include the bone morphogenetic proteins (BMP) for bone formation and platelet-derived growth factor for wound healing [49, 50]. The lack of growth factor release control once the material has been transplanted can result in complications during the use of such materials. Products approved by the FDA generally perform better than preexisting products, but the efficiency of these products varies [36, 51–54]. Most products are, however, unable to fully resolve complex injuries and diseases [36, 52–54]. New biomaterials and stem cell products tend to take time to be introduced into the market mainly due to the number of policies required to get FDA approval and also the lack of monetary funding for these products. Normally, it takes more than 10 years for a product to reach the market whilst more than a billion dollars would have been spent to develop the product [12, 29, 36, 51, 55–57]. Generally, it is much easier and cheaper to introduce a new medical device than it is for drugs and biologics. This has favoured the development of non-cell-based regenerative products than cell-based ones.

One of the most impressive technological advancements of the last decades is 3D printing [58, 59]. Most importantly is the printing of biological material directly onto scaffolds that could be seeded with cells [60]. This is referred to as 3D bioprinting. 3D bioprinting involves different fields including material science, cell biology, and tissue engineering [59, 61, 62]. Successful bioprinting requires the proper placement of biological material, cells, and biomolecules such as growth factors. In order to mimic human tissue, 3D bioprinting must be able to capture the complex structure of the extracellular matrix (ECM) and the different cells present in different tissues [36, 59, 63–65]. In addition, bioprinting must be able to recapitulate the vascular and nervous systems of each tissue needed. In this review, we discuss how recent advances in regenerative medicine and tissue engineering could improve 3D bioprinting and vice versa. We also provide the latest technological advances of 3D bioprinting of potential transplantable tissues and organs. Specifically, we focus on factors required for proper recapitulation of living tissues and organs and their mechanical characteristics and functions. We briefly discuss how this technological advancement is impacting the fabrication of cartilage, heart, and liver.

## 2. Methodology

A literature search from the databases PubMed, Google Scholar, and Science Direct was done from 2000 to October 2017, although we mainly focused on the later years to provide the latest technological advances, for the following key words: biologics, biomaterials, innovation, medicine, native tissue, organs, regenerative medicine, stem cells, tissue engineering, transplantation, and 3D bioprinting. These databases specialize in novel technologies, innovation, human diseases and conditions requiring organ and tissue transplantation, and innovative technologies and mostly use English as the main language. Duplicate articles were removed, and only full articles with the above searched words were included. Articles cited outside of these criteria are to cater for origins of technologies and theories.

## 3. Replacement of Human Body Tissues and Organs

Body tissues and organs have both structure and function, and therefore, any engineered material must be able to recapitulate the morphology and characteristics of the target tissue and organ [66–68]. Several methods have been used to combine both structure and function in engineered tissue or organs. Decellularization of tissues and organs and recellularization before transplantation have shown great promise as they remove immunogenic cells whilst maintaining the structure and material composition of the native extracellular matrix [69, 70]. Decellularization of organs is normally done when the organ is too old to be used for transplantation. Decellularised ECM has also been used as a bioink in 3D bioprinting. Decellularised ECM has the advantage of recapitulating tissue-specific properties and therefore provide the right cues for cellular proliferation and differentiation [69, 71–75]. Issues such as retention of some decellularizing detergent must be addressed. Limitations to this procedure include the use of detergents to wash off all cells so that only the extracellular matrix remains. Cells can then be seeded onto the matrix to restart the process of recellularization. Either stem cells or patient-specific cells can be used in the process. Stem cells are the cells of choice as they can differentiate into several types of cells whereas differentiated cells will only attach and start growing when they find a suitable environment. Together with the use of bioreactors, the approach of decellularization has been used to successfully treat several diseases in animal models [44, 76, 77]. Decellularised tissues and organs can be used as medical devices if the recellularization step is omitted [78–80]. That would shorten the time needed for the product to reach the market as it is considered acellular. There exist several methods of decellularization of tissue and/or organs [36, 42]. Most decellularization methods may affect the mechanical properties of the tissue or organs, and the process may remove signaling molecules, usually tethered to the ECM [36, 42, 69, 71, 74, 77, 81]. If chemicals are used during decellularization, the resulting tissue or organ may transform or degrade over time after transplantation resulting in further complications [44, 69, 72, 77, 82]. It is important to note that other sources of bioink include natural polymers such as starch, dextran, and cellulose.

Synthetic scaffolds do not recapitulate the whole spectrum of properties of native tissue and organs [44, 59, 83, 84]. Most of these scaffolds are fabricated from ECM proteins and also synthetic polymers [59, 85–87]. Hydrogels are especially appealing as they have somewhat similar properties to tissues and are biodegradable [36, 88, 89]. Biodegradability is an important property of hydrogels as it allows the gradual replacement of the hydrogel with a natural scaffold synthesized by cells within the hydrogel and also host cells. The use of hydrogels has been widespread including the treatment of congenital heart defects and in fabricating vascular grafts [36, 62, 90]. Recently, combinations of natural and synthetic biomaterials have been used successfully. This has the advantage of having cell-recognition sites for adhesion and proliferation. Several studies have investigated the proliferation of cells such as chondrocytes in elastin alone and combined with polymers such as polyethylene glycol and polycaprolactone [91–94]. Other studies have also investigated the effect of combinations of ceramics and natural biomaterials such as type I collagen on mesenchymal stem cell differentiation [39]. Overall, composite biomaterials or scaffolds can provide specific properties to advance regenerative medicine and tissue engineering biology. The function of the seeded cells is still debatable with some data showing that seeded cells merely induce inflammation necessary for host cells to populate the graft to form new blood vessels [36, 62]. With this in mind, several vascular grafts, decellularised after the synthesis of the extracellular matrix, are currently under clinical trial [78–80, 95]. The advantage of using such grafts is that they contain an imprint of cells with cues to make more tissue-specific ECM proteins. Several sets of data show that the mechanical properties of the hydrogels and decellularised ECMs do produce a therapeutic and differentiation effect [41, 51, 96–98]. Several studies are underway to elucidate the effect of combining different scaffolds for an additive or enhanced scaffold performance [99–101]. With the advent of noninvasive imaging technologies, it is now possible to create patient-specific replacement tissues based on the patient's body measurements [102]. Such technologies include magnetic resonance imaging (MRI) and computed tomography (CT). Such next-generation imaging technologies have already been used to create patient tailor-made scaffolds. Computed tomography images were used to make a patient's trachea and several other tissues from polymers [103, 104].

## 4. 3D Bioprinting

One major challenge associated with populating scaffolds with cells is the uncontrolled placement of cells. 3D bioprinting has revolutionized the mixing of scaffolds and cells as it can result in structures with some control over material and cell placement in grafts and constructs [102, 105, 106]. 3D bioprinting strategies currently in use include the inkjet, microextrusion, and laser-assisted printing methods. Droplets of scaffolds or hydrogel containing cells are sprayed in the inkjet bioprinting method whilst a continuous stream of ink or scaffold containing cells is dispensed onto a stage in the microextrusion method [31, 107, 108]. These 3D bioprinting methods have resulted in the fabrication of several 3D tissues including cartilage, aortic valves, and blood vessels, with placed cells able to produce ECM proteins such as collagens and fibronectin [36, 109]. Several bioprinting machines have been manufactured, and these have different capabilities [59, 110]. Challenges still remain, however. One major drawback of 3D bioprinting is the low viability of placed cells [111].

In a recent study, Huang and colleagues showed that a graphene-polyurethane nanocomposite hydrogel is a possible bioink for 3D bioprinting of tissue constructs laden with cells [112]. The hydrogel maintained its shear thinning behavior and retained positive effects of graphene or graphene oxide on neural tissue regeneration [112]. Another major drawback of 3D bioprinting tissues is the lack of vascular tissues, resulting in the death of cells due to lack of nutrients and oxygen [113]. Miller and colleagues printed a rigid 3D filament network of carbohydrate glass which they used as a template to generate cylindrical networks that were lined with endothelial cells and extracellular matrices [113]. The perfused vascular channels even sustained the metabolism of rat hepatocytes in tissue constructs [113]. The carbohydrate glass mixture had good enough mechanical stiffness to support its own weight and rapidly dissolve and can be used with cells [113]. Kizawa and colleagues used a scaffold-free 3D bioprinting technology from Cyfuse Biomedical (NA1002, Cyfuse Biomedical) to produce functional 3D-bioprinted liver tissue that was able to maintain glucose and lipid metabolism [114]. The human 3D-bioprinted liver construct also maintained the expression of many drug transporter proteins and metabolic enzymes for many weeks [114]. Such bioprinted liver constructs can be used to predict toxicity in humans [114]. Wang and coworkers presented the design of a low-cost stereolithography system that uses visible light cross-linkable bioinks and produced vertical 3D structures that maintained cell viability for days [115]. This system is likely to be used in tissue engineering and for cell patterning in bioengineering [115]. Graphene-based nanoparticles are especially exciting because they have a high specific surface area and have much better chemical stability [116]. In addition, graphene-based materials can be functionalized and can be used to induce stem cell differentiation and growth [116]. Studies on graphene and its associated nanoparticles are relatively new and will continue to offer important properties that can be exploited in regenerative medicine and tissue engineering. Some of these properties include biocompatibility and specific inductive capabilities [116].

To mimic the complex nature of tissues and organs, there is a need to understand the composition and spatial organization of the components that make up the tissue or organ. Noninvasive imaging technologies such as CT, computer-aided design (CAD), and MRI are being used to provide important information to help design complex tissues and organs [95, 117–121]. Computed tomography shows slices of the tissue architecture that eventually shows the true volume of the tissue and organ under study [95, 117–121]. MRI uses nuclear magnetic resonance and is more powerful in showing enhanced contrast resolution. Computer-aided design and computer-aided manufacturing (CAM-CAD) combined with the use of mathematical modelling techniques can generate 3D models of both tissue and organs [122, 123]. Important properties such as mechanical and biochemical properties can also be predicted through the use of computer-based models. Simulation and structural design of a patient's own organ can also be achieved nowadays. Three main technologies are used in bioprinting of materials of biological origin. These are inkjet printing sometimes called drop-on-demand printing and microextrusion printing where a microextrusion head is used for the printing onto the scaffold and is done by a robot and laser-assisted printing, where laser pulses are used to generate bubbles under pressure, and this sprays the bubble onto the scaffold [59, 124]. A detailed description of these printing technologies is beyond the scope of this review.

### 4.1. Inkjet Bioprinting

**Figure 1:**
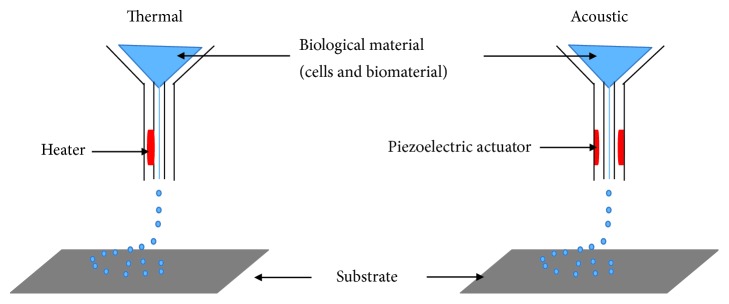
Inkjet bioprinting components. Thermal inkjet printers heat the print head electrically to produce pressure pulses that force droplets of biological material through a nozzle. Acoustic inkjet printers use pulses generated by piezoelectric pressure to break liquids into droplets.

Sometimes referred to as drop-on-demand printers, inkjet printing can be used for both biological and nonbiological applications. Commercially available inkjet paper printers were basically converted into printers of biological material [31, 107, 124–126]. Volumes of biological material in liquid form are sprayed onto defined surfaces with increased resolution and precision and at high speeds. Liquids are ejected from the printer using thermal or acoustic forces onto a scaffold or substrate which is usually part of the graft that will be transplanted onto the tissue (Figure 1). In the case of thermal inkjet printers, a heated print head releases drops of biological material onto the scaffold [108, 127, 128]. The heating does not affect the quality or integrity of the biological material. Thermal inkjet printers are the cheapest of the three bioprinting techniques and are used widely. Inkjet printers are also compatible with many biological materials. Acoustic printers have a piezoelectric crystal that generates acoustic waves [129]. The size of the droplet of the biological material can be controlled by adjusting the duration and amplitude of the wave generated in the printer head. It is very easy to control the size of the biological material droplet as well as the direction of ejection using acoustic inkjet printers. One of the drawbacks of using inkjet printers is the need to maintain a certain viscosity of the biological material being printed [59, 105]. Above certain viscosities, the printer head can be clogged. To maintain biological materials as liquids, usually the number of cells included and therefore printed is lowered. High cell concentrations can jeopardize droplet formation and increase the chances of printer head clogging [59, 130]. So far, inkjet bioprinting has been used to regenerate functional skin and cartilage [92, 131].

### 4.2. Microextrusion Bioprinting

**Figure 2:**
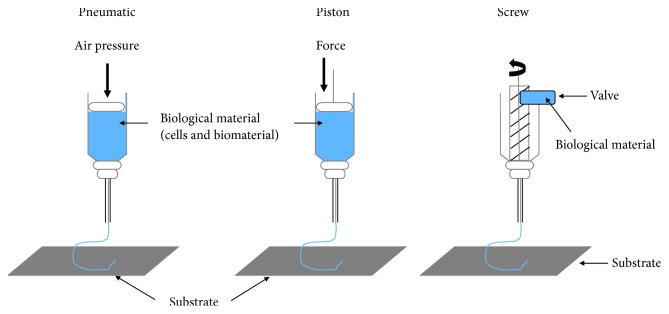
Pneumatic and mechanical (piston and screw) systems are used in microextrusion printers.

Many researchers now use microextrusion technology in tissue and organ engineering studies. Extrusion of biological material through a microextrusion head onto the scaffold or substrate is done by a robot [59, 132]. In this case, continuous small beads of biological material are deposited onto the scaffold as directed by software such as CAM-CAD [59]. Several biological materials can be used with microextrusion printing such as hydrogels and cellular spheroids [133–136]. Two common methods used to extrude biological material are pneumatic and mechanical (piston or screw) (Figure 2). Compressed air is used to force the biological material out through a nozzle at a rate determined beforehand in the pneumatic system [59, 137]. A screw or piston is used to dispense biological material in the mechanical system [58, 124, 137–139]. It is easier to control the flow of material with the mechanical dispensing system than the pneumatic system [59]. For materials with high viscosities, both screw-based and pneumatic systems are the best [140–142]. Several biological materials are compatible with microextrusion printing, and these materials can have a range of viscosities. Unlike inkjet printing, microextrusion bioprinting can be used with high cell densities and therefore achieve cell densities similar to those found under physiological conditions. Microextrusion bioprinting can also print cellular spheroids, and these can then self-assemble into several 3D structures [143, 144]. Scientists believe that cellular spheroids have the same properties as tissue ECM. Vascular tissue spheroids have been generated using the self-assembly of spheroids in 3D-bioprinted organs [145, 146]. One major drawback of microextrusion bioprinting is the lower cell viability compared to inkjet printing. Several tissues have been fabricated using this technique including heart valves, vascular networks, and tumor models.

### 4.3. Laser-Assisted Bioprinting

**Figure 3:**
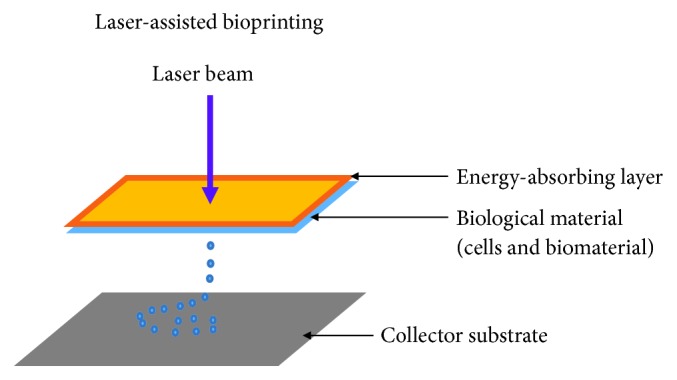
Laser-assisted printers are made up of a pulse laser beam which is focused on an absorbing substrate resulting in the generation of a pressure bubble that forces biological material onto the collector substrate.

Several biological materials including peptides, cells, and DNA have been printed using laser-assisted bioprinting [147, 148]. This method is less commonly used than inkjet and microextrusion bioprinting. In this method, laser pulses are used to generate bubbles under pressure and this sprays the bubble onto the scaffold or substrate (Figure 3). There is no printer head clogging with this method as there is no nozzle. In addition, a range of viscosities can be used with the method. That means cell densities similar to those in physiological tissue can be achieved with minimum effect on cellular viability and function [149]. During printing, there is generation of metallic residues that are present in the final bioprinted material; this contamination constitutes a major drawback of the method [59, 150]. Furthermore, this method is very costly and the hope is that over time these costs will decrease. The capability of laser-assisted bioprinting has been shown in developing several animals and human tissues [151, 152].

## 5. Novel Considerations in Regenerative Medicine and Tissue Engineering

Several factors such as the biomaterial to be used and the cellular source must be considered during tissue or graft manufacture [46, 59]. Such considerations will allow for proper cell-cell and cell-biomaterial (cell-matrix) interactions, thus enhancing the function of the scaffold. Regenerated tissue for transplantation must recapitulate normal tissue in having a specific cell type, with a specific function [58, 138, 139, 153, 154]. Just as in normal tissues and organs, different cells play different roles such as providing structural and supportive roles as provided by endothelial cells. Thus, the cells used during 3D bioprinting will determine the function of the resulting graft or scaffold [44, 59].

The integration of the transplanted graft or scaffold requires that it must self-renew and maintain homeostasis [29, 155, 156]. The most desired source of cells are autologous cells to avoid a host immune response [155, 156]. Autologous cells can be passaged *in vitro* and induced to differentiate into the desired cells before the 3D bioprinting process or transplantation. Several drawbacks are associated with the use of autologous cells. These include the limited regeneration capacity of primary cells and the technical restrictions to the *in vitro* culture of cells. 3D bioprinting is considered more manageable than acellular printing which would require seeding of cells after printing. For grafts to successfully become integrated within the body, there is need for proper integration with the patient's vasculature [157, 158]. Cells in the body are situated near blood vessels to allow for the exchange of nutrients and oxygen [159]. Traditional methods such as biomimetic scaffold fabrication or designing of tissues and organs are unsuccessful when it comes to fulfilling the need for blood vessels and nerves in tissues and organs. Several angiogenic growth factors including VEGF, bFGF, and PDGF have been used in engineered tissues to stimulate blood vessel formation [159, 160]. These growth factors are presented to the scaffolds, and this stimulates the body to initiate angiogenesis. The challenge with the use of growth factors is their short half-lives and their potential for toxic effects [96, 161]. Continuous release of growth factors has been shown to reverse necrosis in some tissues [96, 162]. Prevascularisation of grafts before transplantation is one way to promote graft vascularisation. During 3D bioprinting, endothelial cells can be added to an appropriate material and then transplanted. Several techniques including microfluidic and micropatterning techniques have been used to make or induce the synthesis of vascular networks in tissues [59, 163, 164]. Prevascularisation of the target site has been observed to improve the integration of the transplanted graft [165, 166]. Several tissues will also require the presence of nerves to function properly. Such tissues will require innervation of the grafted tissue by the host for proper integration [59, 167]. Again, growth factors play an important role in stimulating the sprouting of nerves in grafted tissues [168]. In this regard, hydrogels can be patterned with channels loaded with ECM proteins and growth factors to guide nerve formation after transplantation [169, 170].

There are several issues that need improvements regarding cells used for 3D bioprinting. There is a need for cells to survive the actual 3D bioprinting process, remain robust, and continue proliferating and be able to differentiate as in the case of stem cells [59]. Once the scaffold or graft has been transplanted, there is need for cells to have the same cellular function as normal cells. Lastly, all cells used during the 3D bioprinting process must be able to interact directly or through release of biomolecules such as growth factors and cytokines. Cells that can self-renew and have the capacity to generate multiple other cells such as embryonic and adult stem cells are therefore appealing. Adult stem cells are considered safer to use for transplantation than any other cells and remain robust after 3D bioprinting [59, 171]. The presence of exogenously added cells induces a reaction from the host tissue through the secretion of biomolecules including growth factors. Transplanted cells, with or without the scaffold or material, can initiate a response from the host that can heal damaged tissues [172, 173]. Transplanted cells can alter the host ECM composition through secretion of growth factors or synthesis of new ECM proteins or via the secretion of ECM-degrading enzymes such as matrix metalloproteases (MMPs) [174, 175]. The transplanted cells need not be in contact with the host's cells to illicit such a therapeutic response [59, 171, 176]. Mesenchymal stem cells (MSCs) are the cell type of choice when regeneration of damaged tissue is paramount [171, 177, 178]. These cells are thought to be relatively safe compared to embryonic cells. In addition, adult tissue-derived cells are readily and abundantly available. Most therapies available commercially are based on adult tissue-derived cells [69, 179, 180]. Induced pluripotent cells (iPSCs) and embryonic stem cells are potentially abundantly available cells for regenerative medicine strategies [44, 181]. Embryonic stem cells generate all other cell types in the human body, and several studies have established that they are safe for use in regenerative medicine strategies [182, 183]. iPSCs can be obtained from a patient's own cells and therefore raise no issues regarding rejection of transplanted cells [184, 185]. Cells transplanted together with a scaffold are, however, rapidly cleared from the host tissue, and this has the negative effect of limiting their efficacy [186]. To overcome this problem, cells can be encapsulated with material such as hydrogel, and this can lead to a prolonged presence of the cells within the grafted tissue and possibly prevent rejection [187, 188]. By coating transplanted cells with specific antibodies and peptides, such cells can home in to specific tissues and organs [189, 190]. Although the immune system is involved in rejecting grafts or new tissues, it can actively promote the regeneration of damaged tissues as well as enhance engraftment of transplanted grafts [191]. Technological advancement means that the alteration of scaffold characteristics can minimize graft rejection and encourage graft tolerance [191, 192].

## 6. Biomaterials and Cell Interactions: Impact on 3D Bioprinting

In order for biomaterials to be used successfully, specific structure-function relationships must be evaluated between cells and the biomaterials. The most appealing aspect of using synthetic polymers lies in the ability to control cellular microenvironments [193, 194]. Hydrogel mechanical properties such as elasticity and loss moduli are easily changed through the level of cross-linking and do affect cellular growth and differentiation [195, 196]. Furthermore, the inclusion of biomolecules into hydrogels is achieved by simply adding proteins such as fibronectin, collagen, and matrigel to the scaffold [197, 198]. Lately, 3D bioprinting has added a new and innovative dimension to the production of scaffolds for tissue engineering [199]. Beside the inclusion or embedding of biomolecules such as growth factors, cytokines, and chemokines, small molecules that can enhance cellular growth and signaling are now being added routinely to scaffolds [200, 201].

Hydrogels are able to mimic most soft tissues in the human body [196, 202]. Most soft biomaterials are based on natural polymers and their derivatives and also synthetic materials. Examples of naturally occurring polymers include collagen, gelatin, fibrin, and chitosan and are mostly isolated from human or animal tissues [197, 199, 203]. Synthetic polymers include polyethylene glycol (PEG) and pluronic F127. Natural polymers are similar to human ECM and are associated with immunogenic reactions [197, 199, 203]. Synthetic polymers can be tailor-made for the specific tissue or organ. Natural polymers are widely used for 3D bioprinting and do contain a great deal of bioactivity as they contain biochemical cues that drive cell proliferation and differentiation. Hyaluronic acid has been used for the treatment of arthritis and damaged joints [204, 205]. One major drawback of hyaluronic acid is that the hydrogels formed are too soft and swell a lot. The high cost of collagen together with its weak mechanical strength limits its use in 3D bioprinting applications.

Biomaterials have to fulfil certain criteria in order to be used for transplantation. When done *in vitro*, the resultant patch of tissue must be ready for transplantation with the correct properties similar to those of the intended tissue or organ [43, 206]. Different biomaterials can be used as a scaffold to support cell growth and attachment. *In vitro* cultivation of tissues requires cues or signals, usually incorporated into the biomaterial, to enhance cell growth and tissue formation [49, 96, 207–210]. In addition, the biomaterial can be biodegradable so that over time it disappears with its place taken by newly synthesized tissue [211]. Biological cues or signals, alone or incorporated into biomaterials, can be supplied to the body to stimulate tissue regeneration [73, 74, 92, 212–215]. The traditional development strategy for biomaterials involves designing the material composition, modification, and cellular composition followed by *in vitro* evaluation. *In vitro* evaluation looks at parameters such as cellular attachment and growth within the biomaterial, thus impacting on biomaterial modifications and designing [31, 36, 154, 216–218]. *In vivo* evaluation involves testing for biocompatibility with the host tissue as well as efficacy in the host tissue. Finally, clinical testing in human patients is done. Currently, the chances of biomaterial failure are very high though information gained during clinical testing can be used to improve the process of biomaterial design. Such feedback from clinical testing will inform designers about therapeutic processes initiated by the biomaterial. The use of biomaterial, especially the new and technologically advanced biomaterials, to stimulate tissue regeneration is much simpler and straightforward than the use of cells and biomolecules [219]. The gaining of biological function by biomaterials complicates their registration and definition [43]. A balance must, however, be achieved between complexity of biomaterial and efficacy *in vivo*. Designing biomaterials has reached a defining stage, and new considerations such as the relationship between biomaterials and immune system are now discussed.

Biomaterials can have biological signals incorporated within to enhance cellular growth and differentiation [91]. The functionalization of biomaterials is commonplace nowadays and has resulted in the production of several functional tissues [220]. The possession of bioactivity makes a scaffold much more ideal at controlling cellular processes than one that is not functionalized. One way to functionalize scaffolds is by mixing it with growth factors [220, 221]. These biomolecules can then be released slowly to control cell growth and proliferation. Other biomolecules can also be incorporated into the scaffolds such as adhesion molecules and enzyme recognition sites. One major drawback of scaffold biofunctionalization is its effect on the physical and chemical properties of the final scaffold [220]. The addition of adhesion molecules has been reported to increase cellular attachment and even regulate the differentiation of cells. Several types of scaffolds such as hydrogels have been used to achieve sustained release of biomolecules and bioactive components. Incorporated biological signals can also be released at specific stages of tissue regeneration to coincide with specific processes [221, 222]. Cells can also be incorporated in biomaterials with the sole aim of producing biological cues to direct host cellular growth and differentiation [223]. Thus, biomaterial design now focuses not only on providing the physical support needed by cells to grow and for attachment but also on enhancing biological signal production and the delivery of such cues at a specific time during and after transplantation [221, 224, 225]. Importantly, the latest research on biomaterials is now evaluating how biomaterials can regulate immune cell behavior so as to control or diminish immunological reactions associated with tissue or organ rejection [226].

As metals and plastics used in biomaterials come in contact with tissues, there is usually a cellular and immunological response [227, 228]. Several studies investigated the role of macrophages in the inflammatory response associated with the foreign body reaction to the presence of synthetic biomaterials [229, 230]. For biomaterials to integrate into the patient's tissue, there must be some similarity to allow for a seamless interface between biomaterial and surrounding tissue [231, 232]. Biomaterials used in 3D bioprinting must be able to support cellular activity and allow signaling activity between the graft and the host tissue [59, 233]. Any material used in tissue regeneration or graft generation must be controlled and be transferred onto the scaffold or substrate. Inkjet and microextrusion bioprinting methods are limited due to the presence of a nozzle, and therefore, clogging can occur [59, 130, 149]. If the biological material requires cross-linking, this must occur within a short period of time so that more biological material can be added on it. This is especially important for inkjet printing. Some of the latest techniques include 3D powder printing [234–238]. This method uses water or citric acid to bind to the powder or biomaterial into a certain structure [235, 236, 238]. Biomaterials that can be used in this way include starch, gelatin, dextran, and hydroxyapatite [234–238]. They are relatively cheap and do not use harsh conditions, making them usable for biomolecules that are delicate and fragile. They suffer from the need to remove the excess unbound powder at the end of the fabrication process.

The process of building tissues de novo is complex, and therefore, scientists have over time used tissue-derived scaffolds as a starting point. Tissue-derived scaffolds contain instructive signals that direct stem cell differentiation in a certain direction [73, 239]. Several tissues and organs have been decellularised and used as tools for reconstruction of new tissues and organs. Due to the differences in tissue strength and architecture, the decellularization process differs with some tissues or organs requiring more robust methods than others [240]. A balance must be struck between the manipulation of the tissue and organs versus maintaining biocompatibility. The only problematic issue with tissue-derived scaffolds is the lack of knowledge of the chemical and structural composition of the scaffolds, and thus, the origin of the therapeutic effect is not known [241, 242]. Of late, mass spectrometry-based proteomics analysis of the tissue-derived scaffolds has helped identify some proteins and their functions within the scaffolds [243, 244]. Combining ECM proteins with polymers or hydrogels increases the biocompatibility of the resultant scaffold [59, 245]. When implanted into diseased or defective tissue, tissue-derived scaffolds are quickly invaded by cells supporting tissue regeneration. Macrophages are also known to be recruited to the scaffolds where they are responsible for ECM remodeling and debris clearance [246, 247]. The only all-inclusive knowledge needed during biomaterial design can only come from clinical trials although *in vitro* models provide some basic information on the safety of the biomaterial. Parameters such as efficacy can only be studied well *in vivo* as structure-function studies are insufficient. Regenerative methods and the biomaterials used must try to recapitulate the native tissue and its properties [9, 105, 181, 248–250]. This is because one of the challenging factors is integration of transplanted graft and body tissues [95, 119, 251–253]. Induction of healing at the interface of the engineered and native tissue has been suggested as a solution to initiate a healing process. The lack of models to predict human response to the presence of biomaterials and stem cells has been a hindrance in the field of regenerative medicine and tissue engineering [95, 119, 251, 252]. Information from clinical trials has helped improve the design and manufacture of new biomaterials.

## 7. 3D Bioprinting of Tissues

### 7.1. Cartilage Regeneration

Cartilage in the joints provides humans and other animals the ability to move without feeling any pain. Accidents and pathological conditions such as osteoarthritis can lead to cartilage loss and cause painful movements in humans [254–258]. This is because cartilage lines the surface of joints and provides lubrication and “cushions” the body weight during movement. Cartilage is mainly made up of ECM proteins such as type II collagen and aggrecans, and these interact with synovial fluids to provide lubrication and weight-bearing functions [259]. For successful regeneration of cartilage, scientists need to mimic both the surface of the cartilage and its stromal tissue. The use of artificial derivatives from plastics and metals is ridden with disadvantages. For example, plastic and metal implants for cartilage have a short lifespan and can form foreign particles due to wear and tear. Lately, both chondrocytes and MSCs have been used to repair cartilage defects through regeneration [255, 260]. The use of cells, however, gave very disappointing results. This is partly due to the lack of knowledge of the mechanisms involved in cartilage formation. Based on our results, even cell-derived extracellular matrices can direct cells such as adipose-derived MSC differentiation along the chondrogenic lineage [41, 261]. Of late, biomaterials have been developed to mimic the stromal tissue of cartilage and to also promote regeneration of new cartilage. The inclusion of hyaluronic acid in biomaterials and hydrogels has improved lubrication [43, 262]. Most importantly, the inclusion of cells within biomaterials has enhanced the regeneration process and is better than the use of cells and biomaterials individually [41, 258, 263]. Stem cell biomaterial combinations are being evaluated in several translational studies. Several studies have shown that robust soft materials can support the chondrogenic differentiation of stem cells [41, 204, 205]. Several PEG hydrogels in combination with several other polymers are under investigation for repairing cartilage defects [264, 265]. Combinations of alginate, gellan, and type II collagen have all been printed into complex cartilage constructs and showed good biocompatibility and enhanced chondrocyte proliferation [37, 258, 264–267].

## 8. 3D Bioprinting of Organs

The 3D bioprinting of organs is much more complex than that of tissues as it requires the delicate and complex positioning of different cell types in order to recapitulate the natural organ [59, 268]. In addition, organs require the presence of blood vessels and nerves. The question that scientists and clinicians have to answer is whether it is possible to mass produce these complex organs for *in vivo* transplantation. Though successful in the bioprinting of thin tissues, the 3D printing of larger and more complex tissues and organs remains a challenge. Due to their complexity and size, organs tend to take much longer to 3D bioprint, and this has a significant effect on cellular viability [29, 59, 269, 270]. Several biomolecules such as chemokines and growth factors can be added to enhance cellular viability during and after bioprinting. Bioreactors have revolutionized the postprinting process as they can provide the necessary microenvironment needed for long-term storage or culture of the resulting scaffold or graft. Bioreactors recapitulate the natural microenvironment of normal organs in terms of nutrients, oxygen, and biomolecule exchanges. Bioreactors continue to provide the necessary microenvironment for the scaffold or graft to mature over some time [69, 70, 72, 214]. During this period, cells must be able to interact and be able to synthesize the ECM. At the end of the incubation period, an equilibrium must have been achieved between cells, the ECM, and cell surface receptors [66, 69, 250, 271–273]. This will be necessary for the graft or scaffold to be able to integrate with the host tissue. The field of tissue engineering and regenerative medicine has given scientists and clinicians the opportunity to develop full-sized and functional organs for transplantation [66, 69, 250, 272, 274]. The differences in complexities between tissues and organs mean that whereas it is becoming achievable to 3D bioprint several tissues, the bioprinting of organs has remained elusive. Organ-level complexities require the bioprinting of not just one tissue but several tissues and cell lines simultaneously [1, 66, 69, 250]. These tissues and cells must be connected to perform one function. Importantly, tissues must be able to interact with one another and be connected through blood vessels and nerves [168, 275, 276]. Overall, the 3D bioprinting of organs have remained a huge challenge but that has not stopped scientists and clinicians from trying. The generation of mini-organs is definitely a future trend in organ bioprinting.

### 8.1. Heart

The heart is one of the first functional organs to develop during embryonic development [277]. It is needed to pump blood throughout the whole body. The heart is a muscular organ with a very complex structure. The three cells found within the heart are the cardiomyocytes, endothelial cells, and fibroblasts [278]. Heart failure is usually treated via organ transplantation, and with the obvious organ shortages, 3D bioprinting is likely to be a solution to this problem. Several reports show that several heart constructs and grafts are under evaluation [90, 279–282]. The heart requires proper vascularization and innervation for it to function properly. Therefore, heart constructs and grafts must have adequate vascularization, and this represents a huge challenge. The heart ECM is a major player in cellular differentiation and determination of protein expression. The heart ECM is mainly made up of collagen. Due to its complexity, several approaches including allografts, xenografts, and autografts have been tested. The promise of tissue engineering and regenerative medicine has not gone unnoticed when it comes to repairing the heart and addressing cardiovascular disease. 3D bioprinting has been used so far to engineer functional cardiac tissue and heart valves. Biodegradable biomaterials are usually used for heart valve bioprinting and can still mimic the valvular anatomy. Several 3D bioprinting methods and cells have been used to print heart tissue that beats [44, 59]. Embryonic stem cells can form embryoid bodies [98], and laser direct write bioprinting can control the size and formation of embryoid bodies [283, 284]. MSCs and endothelial cells have also been printed onto a patch, thereby promoting blood vessel formation [31, 59, 283, 284]. Most 3D-bioprinted cells retained their high cell viability and differentiation towards cardiac lineage as determined by cardiac transcription factor gene expression. Coronary artery blockages or myocardial infarction causes serious damage to the heart, and engineered myocardial tissue has been studied as a replacement [285, 286]. Myocardial infarction results in heart failure mainly due to cell death through necrosis. Indeed, bioprinting processes have been used to make viable patterned patches allowing for the improvement of infarcted hearts after transplantation. For example, an alginate hydrogel loaded with cardiomyocyte progenitor cells maintained cell viability and increased the healing of the heart tissue. Decellularised heart tissue has been used in microextrusion bioprinting to create heart tissue [8, 287]. In addition, the bioprinting of living prosthetics that can adjust to the heart condition and integrate better to the human heart than nonliving prosthetics has resulted in enhanced performance of the prosthetics.

### 8.2. Liver

Most of the liver tissue is made up of hepatocytes [288]. Several other cells such as portal fibroblasts and endothelial cells are also found in the liver. The liver is involved in many important metabolic processes such as plasma protein synthesis, hormone production, and detoxification of xenobiotic compounds. The liver consists of four hepatic lobes and two major cells existing in the liver, the parenchymal and the nonparenchymal cells. The hepatocytes have a high regenerative capacity making the liver one of the organs with high regeneration capacity. Hepatocytes, however, functionally deteriorate fast once maintained *in vitro* [289]. Adult stem cells are the best choice for 3D bioprinting of hepatic tissue since they can be obtained from the patient, allowing personalized tissue bioprinting [173, 289, 290]. Stem cells also express hepatocyte-like genes. The fabrication of microlivers has allowed the study of several candidate drugs in high-throughput studies. 3D hepatic tissues have been developed using bioprinting techniques [291, 292]. Embryonic stem cells have been bioprinted using valve-based bioprinting to create liver constructs, and the cells differentiated to be hepatocyte-like cells [36, 181, 293]. The cellular sources used in liver constructs or grafts include adipose-derived stromal cells, Wharton-jelly derived stromal cells, and hepatic progenitor cells. Bioprinted cells demonstrated hepatocyte-like phenotypes such as secretion of albumin. The complexity of these constructs was further enhanced through the addition of endothelial cells. Hydrogels made up of different combinations of gelatin, polyethylene glycol, and alginate have been used to 3D bioprint liver-like constructs [32, 105, 248, 289-291, 294–298]. Most 3D-bioprinted tissues show liver-specific functions in addition to injury response. Several companies and research groups have created liver constructs mimicking native liver structures and functions [52, 289, 290, 292, 299, 300]. There is an acute demand for livers, and the fabrication of liver tissue or the liver will definitely alleviate this problem. Liver tissue and organoids can also be used in other assays such as drug testing and liver disease studies. As with mature hepatocytes, hepatocyte-like cells obtained from stem cells tend to functionally deteriorate fast under *in vitro* conditions [289]. The liver structure is complex with a modular microenvironment; thus, it is difficult to model native liver tissue [289].

## 9. Medicinal Remedies in Regenerative Medicine and Tissue Engineering

**Figure 4:**
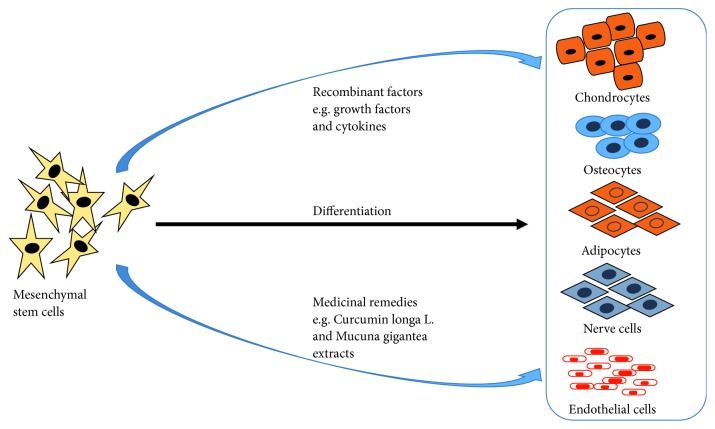
Stem cells such as mesenchymal stem cells can be differentiated through the use of synthetic factors and/or medicinal remedies into different cell types. Medicinal remedies have the advantage of causing less side effects and being very cheap.

Most 3D bioprinting processes and stem cell therapies require the use of synthetic and natural biological molecules such as growth factors to enhance the proliferation and differentiation of stem cells [155, 301]. Reports of severe side effects and toxicity from the use of these substances have surfaced, and scientists are searching for alternatives. Most of the current stimulants are of nonhuman origin and therefore may be rejected when used. In addition, the use of these purified biological molecules is an expensive option. The need to replenish growth factors during stem cell differentiation, due to their short half-lives, make their use an expensive option, especially in developing countries [302, 303]. Medicinal or herbal plants are used mostly in the developing world for primary health care. The last 5 years has seen an increase in the use of medicinal plants for health promotion and treatment of diseases in developed countries [304, 305]. Indeed, many medicinal plant extracts are now used as prescription drugs in many developed countries such as the United Kingdom, Germany, and France [306, 307]. Our data show that resveratrol treatment upregulates collagen type II in chondrocytes and can increase chondrocyte viability [308]. Resveratrol, therefore, can be used during 3D bioprinting of cartilage constructs to enhance chondrocyte viability after the printing process. Ethanol and dichloromethane extracts of *Pleurostylia capensis* Turcz (Loes) were shown to have antimicrobial, antioxidant, and anti-inflammatory activities [308, 309]. Many medicinal plant extracts have shown anticancer activities through inhibition of cancer cell proliferation and growth. Of late, several medicinal plant extracts have been used to promote stem cell proliferation and differentiation and to encourage tissue regeneration leading to rehabilitation of damaged or diseased tissues (Figure 4) [310–312].

Several studies have been undertaken to study the effect of medicinal plant extracts on stem cell differentiation with the hope of providing nontoxic and affordable stem cell therapy and tissue and organ transplantation [310–312]. Promising results have been shown in the treatment of several pathological conditions such as osteoporosis, neurodegenerative disorders, and degenerative ailments using medicinal plant extracts [307, 313–317]. Medicinal plant extracts are an affordable and readily available option since they have been in use since time immemorial. The only drawback on the use of medicinal extracts is the lack of knowledge on the mechanism of action of these extracts. Issues such as variability, toxicity, and complexity of the medicinal extracts have limited their clinical use in stem cell therapy and tissue engineering procedures [314–317]. It is hoped that once purified and standardized, medicinal extracts can be used in many applications requiring tissue regeneration and enhanced stem cell growth. In addition, understanding the mechanisms and signaling pathways involved in medicinal extracts' healing potential or power is a necessity before they can successfully be used in 3D bioprinting and regenerative and reparative therapies [314, 316, 317]. The advantages of using these medicinal extracts stem from their availability, low cost, and nontoxicity if taken in certain doses [314–317]. Most of these extracts are already in use to treat several ailments.

The health benefits of using plant-based remedies are known to include the prevention of certain ailments such as headaches and the common cold. Most medicinal plant extracts are used as cocktails, and the combination of different phytochemicals is thought to have an additive or synergistic effect on different pathological conditions [310–312]. Several medicinal plant extracts have been suggested to stimulate adult stem cell proliferation and thus regeneration of damaged or diseased tissues. A study by Kim and colleagues showed that Aconiti Lateralis Preparata Radix (ALR) promoted mouse bone marrow-derived mesenchymal stem cell proliferation by more than 100% compared to controls [318]. Several studies also showed that blueberry and catechin all have a dose-dependent effect on human bone marrow proliferation compared to the granulocyte-macrophage colony-stimulating factor [319–321]. Several plant extracts were shown to increase the healing of scratch wounds in several assays compared to controls [322, 323]. Polysaccharides and hyperforin from the medicinal plant *Hypericum perforatum* also known as St John's wort stimulated the differentiation of keratinocytes in several studies [324, 325]. Extracts from two Chinese medicinal plants, *Angelica* and *Chuan Xiong*, showed significant angiogenic effects and could be of use during the treatment of myocardial infarction and peripheral ischemia [326].

A component of *Rhizoma drynariae* extract, naringin, has been shown to increase osteogenic differentiation of bone marrow-derived MSCs [327, 328]. An extract from another medicinal plant *Herba epimedii* also enhanced osteogenic differentiation of bone marrow-derived MSCs, and this activity was mainly due to flavonoids in the extract [327–329]. A fraction of Dipsaci Radix also enhanced osteoblastic differentiation of bone marrow-derived MSCs [330, 331]. Acetic acid extracts of *Mucuna gigantea* promoted the proliferation of bone marrow-derived MSCs as well as the expression of neural markers nestin and *β*-III tubulin. It was also shown that the *Mucuna gigantea* extract contains L-DOPA, a precursor of dopamine. The use of *Mucuna gigantea* extract to promote nerve formation during stem cell therapy is appealing. Adipose-derived MSCs treated with *Radix angelica sinesis* extract showed increased neural like cell differentiation compared to cells treated with butylated hydroxyanisole, a commonly used neuronal inducer. Another important component of *Radix angelica sinesis* extract, ferulic acid, was shown to decrease neurotoxic *β*-amyloid peptide aggregation in several animal models [332, 333]. An extract from another medicinal plant, *Salvia miltiorrhiza*, induced neurogenic differentiation of Wharton's jelly-derived MSCs with significant upregulation of markers such as nestin, a glial fibrillary acidic protein [334, 335]. Curcumin is a major component of *Curcumin longa* L. extract and has anti-inflammatory properties. An ethanol extract of *Curcumin longa* L. induced endothelial differentiation of adipose-derived MSCs [336]. The commonly used olive leaf extract induced endothelial differentiation and formation of tubular structures in mesenchymal stem cells, suggesting it is important for blood vessel formation [337, 338].

Thorough research on the use of medicinal plant extracts in 3D bioprinting, regenerative medicine, and tissue engineering is needed so as to understand the mechanisms and signaling pathways involved before they can be used successfully in fields. One major drawback is the solvents used to extract the compounds. Some of the solvents such as methanol and acetic acid may cause undesirable effects when used during transplantations and therapy. Most extracts are not similar, displaying a variability with each extraction. In addition, most extracts are mixtures of many compounds that might require purification before they can be used.

## 10. Challenges to 3D Bioprinting of Tissues and Organs

Due to 3D bioprinting being an interdisciplinary field, it will require teams of scientists from different fields to come together to make it successful. There are many challenges that need to be addressed before we can advance the few available proof-of-concept examples into real tissue and organ 3D bioprinting. At the moment, the designing and fabrication of tissues and organs require standard methods [44, 59, 85]. This is difficult given that some of the cells are obtained from individuals who differ remarkably from each other. Thus, the way the cells will eventually proliferate and differentiate will be different. Many technical challenges must be addressed as well. These include the lack of speed during the bioprinting process as well as the biocompatibility of the materials used [31, 58, 137, 236]. In addition, several tissues require the presence of different biomaterials and cells. These will have to be printed at the same time in precise locations within the graft or scaffold. This might require a combination of different bioprinting strategies. Post-bioprinting, the scaffold or construct must be cultured for some time in a bioreactor for maturation [69, 71, 159, 272, 339]. This is needed to allow cells to deposit the ECM and synthesize biomolecules such as growth factors needed for a living construct.

One of the major challenges in regenerative medicine and tissue engineering that is addressed by 3D bioprinting is vascularization [144, 340–344]. Several studies have been done and successfully created 3D vascularised tissues in animals and human tissues [345–350]. Arkudas and colleagues showed that vascularised constructs used for femoral defects in rats and in sheep led to increased bone formation [351, 352]. If successful, 3D bioprinting can also be personalized to suit the needs of a particular individual. Considering the above, high-quality 3D bioprinting is necessary so that the resulting construct or graft can be used in humans. Each step along the way will require stringent quality controls consistent with drugs used for humans. Most trials so far have been done in animals. Finally, all constructs and grafts will have to be approved by the relevant authorities such as the FDA or the European Medicines Agency. Although challenges remain, the field of tissue engineering and regenerative medicine has great potential and this will only be realized if scientists and clinicians can work together to advance the bioprinting techniques and engineering designs. 3D bioprinting has such an appealing versatility that it can also be expanded to include the development of tissues and organs for other research areas such as drug toxicity and oncology.

## 11. Conclusion

A number of diseases and conditions are now being treated via the use of regenerative medicine. The continual manipulation of both scaffolds and cells will allow for the control of the host's response to the presence of the scaffold and cells in 3D-bioprinted constructs or organs. Technological advances will allow for the fabrication of patient-specific and tailor-made grafts that will position cells within specific regions of the scaffold and possibly mimic native tissues. Most importantly, graft integration with host tissue will improve with new knowledge on graft vascularization and innervation. Improved techniques with regard to the release of growth factors within 3D-bioprinted constructs and organs once transplanted will allow the controlled healing and regeneration process. Modulation of the immune system can lower the rejection of 3D-bioprinted tissues and organs or at least allow scientists to achieve a desirable immune response. Increased knowledge on stem cell behavior and controlled differentiation of the cells can be achieved, allaying fears of their safety. Alteration of the host environment to prevent rejection of 3D-bioprinted constructs and organs and to provide the right niche for the transplanted cells will allow cells to grow under their “normal conditions,” thus improving outcomes of regenerative medicine strategies. The latest research also points to the microbiome affecting almost all cellular processes of the body; thus, knowledge of the role the microbiome plays in construct or graft integration is important. 3D-bioprinted models of human diseases and conditions must continue to improve to allow for the translation of promising regenerative medicine strategies. The future of regenerative medicine and tissue engineering relies on the ability of scientists and clinicians to “mimic nature” or “work with nature” in coming up with innovative biomaterials and technologies such as nanotechnology to advance this field.
